# The Complete Sequences and Ecological Roles of Two IncP-1β Plasmids, pHB44 and pBS64, Isolated from the Mycosphere of *Laccaria proxima*

**DOI:** 10.3389/fmicb.2016.00909

**Published:** 2016-06-21

**Authors:** Miaozhi Zhang, Jolanda K. Brons, Jan Dirk van Elsas

**Affiliations:** Department of Microbial Ecology, Center for Ecological and Evolutionary Studies, University of GroningenGroningen, Netherlands

**Keywords:** IncP-1β plasmids, horizontal gene transfer, plasmid evolution, mycosphere, *Variovorax paradoxus*

## Abstract

Two novel plasmids, coined pHB44 and pBS64, were recently found in *Variovorax paradoxus* strains HB44 and BS64 isolated from the mycosphere of *Laccaria proxima*, on two different sampling occasions. We here describe the full sequences of pHB44 and pBS64 and establish their evolutionary placement and ecological function. Both plasmids, unique for mycospheric *V. paradoxus*, were around 58 kb in size. They possessed, in a very similar fashion, three main plasmid backbone regions, which were predicted to be involved in plasmid replication, central control of maintenance, and conjugational transfer. Phylogenetic inference on the basis of seven selected and concatenated plasmid backbone genes provided solid evidence for the placement of the two plasmids in the IncP-1β1 group, with the recently isolated IncP-1β1 plasmid pMBUI8 as the closest relative. A comparative analysis of the sequences present in each of the recombinational hot spots (RHS) I to III across plasmids pHB44, pBS64, and pMBUI8 revealed the insertions found in plasmids pHB44 and pBS64 to be different from those of pMBUI8. Whereas, in the former two plasmids, RHS I and III were devoid of any major inserts, their RHS II regions contained inserts of 15,043 (pHB44) and 16,406 kb (pBS64), against about 9,3 kb for pMBUI8. Interestingly, these regions were highly similar across plasmids pHB44 and pBS64, and differed from that of pMBUI8. Closer inspection revealed the insert in the former plasmids to contain, next to transposases, an “*mmf*” gene cassette previously reported to encode metal “responsiveness” in the PromA plasmid pMOL98. Whereas the plasmid pHB44 RHS II contained the canonical *mmf* sequence, that in pBS64 contained, in addition, a “two-gene duplicated region” flanking the *mmf* C2 gene. *In vitro* experiments on the growth and survival of strains with or without plasmid pHB44 suggested this plasmid was involved in the binding and import of Fe^3+^ as well as V^3+^ ions into the host cells, thus yielding a growth advantage under “metal ion-limiting” conditions. In addition, pHB44 was found to confer a bacitracin resistance phenotype to its host strain HB44. The metal import and bacitracin resistance traits were tentatively attributed to specific genes present in the RHS II inserts.

## Introduction

Horizontal gene transfer (HGT) is regarded to constitute a crucial mechanism for bacterial adaptation and evolution, as it enables bacteria to exchange genes and thus to adapt in fast-changing environments (Thomas and Nielsen, 2005). Among the HGT mechanisms, conjugative plasmid transfer is likely to be a major contributor to the genetic plasticity of natural bacterial communities (Frost et al., 2005). Plasmids are extrachromosomal units that can self-replicate, and particular subclasses of plasmids encode their own conjugational transfer systems. Moreover, plasmids are often equipped with so-called “accessory” genes that encode host-beneficial functions, such as resistance to antibiotics or heavy metals, degradation of xenobiotic compounds, virulence, and symbiosis (Sota et al., 2007). Comparative studies of conjugative plasmids have revealed that their backbones are often conserved per group, whereas the accessory genes carried are genetically diverse (Dennis, 2005).

The large diversity of plasmids can be addressed using the incompatibilities of their replication systems; thus, so-called “Inc.” groups have been formed. Plasmids from one of these groups, the IncP-1 group, are broad-host-range, implying that they are able to self-transfer and be maintained in a large variety of (Gram-negative) bacteria (Thomas, 2000; Dennis, 2005). IncP-1 group plasmids have been widely isolated from wastewater, sludge, and soil environments (Schlüter et al., 2003, 2007; Tauch et al., 2003; Heuer et al., 2004; Sen et al., 2011). So far, a large consistency has been observed with respect to the plasmid backbone sequences, in addition to often divergent “accessory” genes (Thorsted et al., 1998; Schlüter et al., 2007). The latter genes are usually inserted in RHSs located between the *oriV* and *trfA* genes (RHS I), between the two transfer operons *tra* and *trb* (RHS II) and/or upstream of the *klc*A gene (RHS III; Dennis, 2005). On the basis of molecular typing of selected backbone genes, the IncP-1 plasmids are currently classed in six subgroups, denoted IncP-1α through IncP-1ζ (Norberg et al., 2011). Among the subgroups, the IncP-1β plasmids are of particular interest due to their prevalence in soil environments and the accessory traits that are often encoded by the genes present in RHS regions (Heuer and Smalla, 2012).

The mycosphere is the microhabitat that surrounds fungal hyphae in soil, one particular example being the soil beneath fungal fruiting bodies (Warmink and van Elsas, 2008). Carbonaceous compounds that are released from the fungal hyphae stimulate the growth of heterotrophic bacteria. As a result, the bacterial numbers in the mycosphere (just like in the rhizosphere and mycorrhizosphere) are often higher than those in the corresponding bulk soil. This defines a prime site where bacterial activity occurs, as outlined in Warmink and van Elsas (2008). The mycosphere was also proposed to constitute a soil arena where organism-to-organism contacts are activated and so HGT is stimulated. Thus, genes, including locally-adaptive ones, may be exchanged across the local communities (Zhang et al., 2014a). Indeed, recent evidence from triparental exogenous isolations has indicated that plasmid transfer frequencies are significantly higher in mycosphere-dwelling bacterial communities of several fungi than in those of corresponding bulk soils (Zhang et al., 2014b). Moreover, the densities of IncP-1β plasmids were also significantly increased in several mycosphere soils (Zhang et al., 2014b).

In a recent study, we reported experiments with two plasmids, pHB44 and pBS64, of *Variovorax paradoxus* obtained from the mycosphere of *Laccaria proxima*. Replicon typing provided preliminary evidence that both plasmids belong to the IncP-1β group (Zhang et al., 2015). Also, both were able to mobilize selectable IncQ group plasmids into other bacteria. In contrast to other IncP-1β plasmids, plasmids pHB44 and pBS64 did not contain resistances to any of the antibiotics or heavy metals used. Remarkably, plasmid pHB44 enhanced the fitness of strain BS64 in soil microcosms with the fungus *Lyophyllum* sp. strain Karsten under iron-limiting conditions, indicating a fitness-enhancing role for its host in this setting (Zhang et al., 2015). As pHB44 and pBS64 thus constitute the first plasmids that foster host fitness in the mycosphere, we here broaden the scope of the analyses. In particular, following a search for additional plasmids with relevance for life in the mycosphere, we performed a detailed analysis of the pHB44 and pBS64 nucleotide sequences and their potential host fitness-affecting roles.

## Materials and methods

### Bacterial strains and growth conditions

Twenty-eight *V. paradoxus* rhizosphere-isolated strains were obtained from INRA Dijon, France (Dr. P. Lemanceau) and 15 such strains were isolated by us on nutrient agar plates from the rhizosphere of *Oxyria digyna* growing in Kilpisjarvi, Finland. As reported, the two strains denoted HB44 and BS64 were from the mycosphere of *L. proxima*, Gieterveen, The Netherlands (isolated in 2004 and 2006, respectively; Warmink and van Elsas, 2008). Furthermore, *Escherichia coli* K12 and K12 (pMBUI8) (kindly received from E.M. Top, Idaho, USA), *Pseudomonas fluorescens* R2f as well as *Burkholderia terrae* BS001 were used. All strains were grown in R2A (yeast extract 0.5 g, proteose peptone 0.5 g, casamino acids 0.5 g, dextrose 0.5 g, soluble starch 0.5 g, sodium pyruvate 0.3 g, dipotassium phosphate 0.3 g, magnesium sulfate 0.05 g, distilled water 1 L; pH 7.2) and Luria-Bertani [LB] broth (tryptone 10 g, yeast extract 5 g, NaCl 5 g, distilled water 1 L; pH 7.2), respectively, at 28°C for 24 h. Agar (1.75%) was added to the media when necessary.

### Plasmid extraction and purification

Plasmid DNA was obtained routinely following a modified extraction protocol (Birnboim and Doly, 1979). In short, overnight-grown cell pellets were obtained and resuspended in resuspension buffer, which was followed by adding lysis solution and incubating at room temperature for 5 min. Afterwards, 150 μl of 7.5 M ammonium acetate and 150 μl of chloroform were added and the tube was incubated on ice for 10 min, followed by a spin for 10 min. Later, supernatant was transferred to 200 μl precipitation solution and chilled on ice for 15 min. Following centrifuging for 15 min, the supernatant was removed and the pellet air-dried. Finally, the pellet was resuspended in demineralized water. The quantity and quality of plasmid DNA were checked on 1% agarose gels and verified by ethidium bromide staining. The resulting images were digitized. Bands containing plasmid DNA (around 58 kb for pHB44 and pBS64) were excised from the gel and extracted with the Zymoclean™ Large Fragment DNA Recovery Kit (catalog number: D4045, Zymo Research, USA). Ultrapure plasmid DNA was obtained and sent for sequencing at LGC (Berlin, Germany).

### Plasmid curing

Here, curing was used to produce a plasmid-cured derivative of *V. paradoxus* strain HB44. Strain BS64 had already been cured, as reported before (Zhang et al., 2015). Briefly, we applied serial-batch transfers of the relevant cultures using (1) raised temperature (33 and 37°C) (2) sub-inhibitory concentrations of novobiocin (7 μg/ml) or ethidium bromide (4 μg/ml). After each transfer, in particular focusing on transfers 5, 10, and 20, up to 50 colonies were checked per culture by colony PCR (on the basis of the *trf* A gene; Götz et al., 1996), to assess the putative loss of the IncP-1β plasmid. Potentially cured clones were subjected to plasmid extractions and further testing in order to reveal the absence of the plasmid.

### Restriction analysis of plasmid DNA

Digestion of plasmid DNA was performed in a 100 μL DNA digestion mix, consisting of 10 μL digestion buffer, 4 μL enzyme, and 100 μg of pure plasmid DNA. Sterile water was added to an end volume of 100 μL. Digestion was done for up to 60 min (using EcoRI and SphI) or 2 h (BamH1 and HindIII) at 37°C.

### Sequencing of pHB44 and pBS64 DNA

A preliminary account of pHB44 data produced by a previous sequencing run via Roche 454 FLX pyrosequencing has been given before (Zhang et al., 2015). Some of these (incomplete) sequences supported the current, improved, sequencing effort. Thus, the complete sequences of plasmids pHB44 and pBS64 were obtained as multiple reads. Library generation for the 454 FLX sequencing was carried out according to the manufacturer's standard protocols (Roche/454 life sciences, Branford, CT 06405, USA). In short, for each library the plasmid DNA was sheared randomly by nebulization to fragments ranging in size from 400 to 900 bp. These fragments were end-polished and barcoded. For that, 454 A and B adaptors that are required for the emulsion PCR and sequencing were added to the ends of the fragments by ligation. The resulting fragment libraries were sequenced on a 1/16 pico titer-plate (PTP) on the GS FLX using Roche/454 titanium chemistry. Totals of 30,809 and 35,466 sequence reads with average read lengths of 462 nucleotides were obtained for plasmids pHB44 and pBS64, respectively.

### Assembly and annotation

Contiguous sequences (contigs) > 1.5 kb from the high-throughput (Roche 454) sequencing (LGC Genomics) were initially used to construct draft plasmid genome sequences of pHB44 and pBS64. For plasmid pHB44, the previously obtained sequence information (Zhang et al., 2015) was also used. The contigs were also checked using BLAST to search for the closest-related known (plasmid) gene or region in Gene bank, which was used as a reference for building the plasmid. MEGA software (Tamura et al., 2011) was used to construct the backbones of the plasmids. Several gaps remained, for each plasmid, after these initial analyses. Some were subsequently closed by aligning with the trimmed raw reads (removing 15 nucleotides from both the 5′ and 3′ ends). For the remaining gaps, amplifications were performed on the basis of an educated guess about gap nature, taking reference sister plasmids as the comparator matrix. The PCR products were then sequenced using a ABI3730XL sequencer. This approach resulted in the closure of all remaining gaps in the two plasmids. The draft plasmid DNA sequences were then annotated by using RAST platform (Overbeek et al., 2014). All putative coding sequences (CDSs) were manually checked for quality. Interpro (Jones et al., 2014) was then used to assign functions to the predicted hypothetical proteins. The final circular maps of pHB44 and pBS64 were generated using DNA-MAN software (version 7.212, Lynnon Corp., Quebec, Canada). The sequences are available under numbers KU356988 and KU356987 in NCBI.

### Bioinformatics analyses and software

To understand plasmid evolution, selected plasmid nucleotide and predicted amino acid sequences were compared with those of related plasmids deposited in the NCBI repository by BLAST-N and BLAST-X (Altschul et al., 1997). Synteny analysis was performed using the Mauve Alignment Tool (Darling and Perna, 2010). Repeat regions within the pHB44 and pBS64 sequences were identified and analyzed by using REPuter software (Kurtz et al., 2001). Insertion sequence elements were identified and annotated by using the IS database (https://www-is.biotoul.fr/; Siguier et al., 2006) whereas genomic islands were identified using the RGP_Finder available in the MaGe platform. Tetranucleotide frequencies were calculated using the software JSPECIES (http://www.imedea.uib-csic.es/jspecies/; Teeling et al., 2004a,b; Richter and Rosselló-Móra, 2009).

Multiple sequence alignments were done using ClustalW incorporated in Mega5 (Tamura et al., 2011). Phylogenetic network analysis and the Ø-statistics were carried out using the SplitsTree program (Bruen et al., 2006). The splits network (neighbor net) was constructed using uncorrected P character transformation, which computes the proportion of positions at which two sequences differ (Huson and Bryant, 2006). Finally, bootstrap values were derived from 1000 repetitions.

Accession numbers of the IncP-1 plasmids included in this study are as follows: pQKH54 (NC_008055), pBS228 (NC_008357), pKJK5 (NC_008272), pA81 (NC_006830), pB4 (NC_003430), pA1 (NC_007353), pB10 (NC_004840), pJP4 (NC_005912), pBP136 (NC_008459), pUO1 (NC_005088), pTP6 (NC_007680), pADP-1 (NC_004956), pB3 (NC_006388), R751 (NC_001735), and pB8 (NC_007502), pAKD4 (NC_025029), pMCBF6 (NC_025028), pMCBF1 (AY950444), pMBUI8 (NC_025090.1), pHB44 (KU356988), and pBS64 (KU356987).

### Growth of plasmid-containing vs. plasmid-less strains under iron limitation

To determine the growth of *V. paradoxus* strain HB44 (pHB44) and its plasmid-cured derivative HB44 in different iron concentrations, 100-ml flasks containing 20 ml M9 mineral medium (200 mL 5X M9 salts solution (64 g Na_2_HPO_4_.7H_2_O, 15 g KH_2_PO_4_, 2.5 g NaCl, 5.0 g NH_4_Cl per liter), 2 mL of 1 M MgSO_4_.7H_2_O, 0.1 mL of 1 M CaCl_2_, 10 ml of 100 × (Fe-less) trace element solution (5 g/L EDTA, 84 mg/L ZnCl_2_, 13 mg/L CuCl_2_.2H_2_O, 10 mg/L CoCl_2_.2H_2_O, 10 mg/L H_3_BO_3_, 1.6 mg/L MnCl_2_.4H_2_O), 790 mL of distilled water) were used (Sambrook and Russell, 2001). To these, 1% of glycerol was added as the carbon source. Three levels of added FeCl_3_ were then established in the M9 media, i.e. zero, 5 and 50 μM. Cells from overnight cultures were washed twice and introduced at levels of 10^5^ cells/ml into the media. Growth was monitored for 3 days by periodically sampling the cultures and assessing the CFU numbers by dilution plating, as well as by the OD600 absorbance values.

### Growth of plasmid-containing vs. plasmid-less strains under vanadium limitation

The growth of *V. paradoxus* HB44 (pHB44) and its plasmid-less derivative HB44 was then assessed in M9 minimal medium (see above, 30 μM Fe present in the trace element solution) amended with 0, 5, and 50 μM of VCl_3_. Growth was monitored for 3 days by periodically sampling the cultures and assessing the CFU numbers by dilution plating as well as OD600 absorbance measurements.

### Bacitracin resistance assay

*V. paradoxus* strains HB44 (pHB44), HB44, BS64 (pBS64), and BS64, next to the controls *E. coli* K12 (pMBUI8) (presumed to be similar to our new plasmids) and K12 (negative control), were streaked, as well as diluted, to single-colony-formation on R2A plates supplemented with 0, 10, 20, 40, 80, 160, 320 and 500 μM of bacitracin. In this way, their minimal inhibitory concentrations (MICs) were determined. The plates were incubated at 28 (*V. paradoxus*) and 37 C (*E. coli*) and growth was monitored daily, for a period of up to 10 d. In subsequent experiments in liquid media, strains HB44 (pHB44) and HB44, BS64 (pBS64), and BS64 were grown in R2A or LB broth with bacitracin added under their MIC respectively, and growth rates were determined by determining OD420 values and CFU counts.

## Results

### Intermediate-sized plasmids of the IncP-1 group are “unique” for *V. paradoxus* strains obtained from the mycosphere

Twenty-eight *Variovorax* strains isolated from the wheat rhizosphere as well as 15 strains isolated from the mycorrhized *O. digyna* rhizosphere were subjected to plasmid extractions using the protocol of Birnboim and Doly (1979). As controls, plasmids pHB44 and pBS64 were extracted from their respective hosts *V. paradoxus* HB44 and BS64. The data showed that none of the 43 new strains yielded covalently closed circular (ccc) bands on gels that would indicate the presence of plasmids of between roughly 12 and 80 kb in size, whereas the extractions from strains HB44 and BS64 consistently revealed the successful extraction of ccc DNA of about 58 kb. Moreover, IncP-1 plasmid based replicon typing (Götz et al., 1996) data performed on both the extracts and colony material of all strains were negative for the 43 novel strains, again with positive signals for the HB44 and BS64 plasmid DNAs. This confirmed the contention that sequences of plasmids of the IncP-1 [and IncQ] types are rare in the aforementioned rhizosphere strains (data not shown). Hence, the occurrence of plasmids in the whole set of *Variovorax* strains was “unique” for the mycosphere isolates HB44 and BS64 (yielding pHB44 and pBS64, as reported; Zhang et al., 2015), as compared to those from two divergent rhizospheres.

### Plasmids pHB44 and pBS64 are broad-host-range mobilizer plasmids that can transfer and mobilize IncQ plasmid pSUP104 across a suite of gram-negative bacteria

In previous triparental pSUP104 mobilizations with pHB44 and pBS64 as the drivers (Zhang et al., 2015), we found transfer frequencies from *V. paradoxus* to *P. fluorescens* R2f Rp^r^ (ratio of transconjugants to recipients) for pHB44 and pBS64 in the order 10^−4^ to 10^−5^. Here, we further transferred both plasmids from the *P. fluorescens* R2f Rp^r^ transconjugants to *B. terrae* BS001 Sm^r^, at transfer frequencies of about 10^−5^ per recipient. Both plasmids pHB44 and pBS64 were stable upon growth for >20 generations in the *B. terrae* BS001 Sm^r^ host. Hence, plasmids pHB44 and pBS64 clearly are natural vectors of broad host range gene transfer across the mycosphere dwellers *V. paradoxus* (β-Proteobacteria), *P. fluorescens* (γ-Proteobacteria), and *B. terrae* (β-Proteobacteria).

### Plasmid genomes—general sequence features

**Figure 1:**
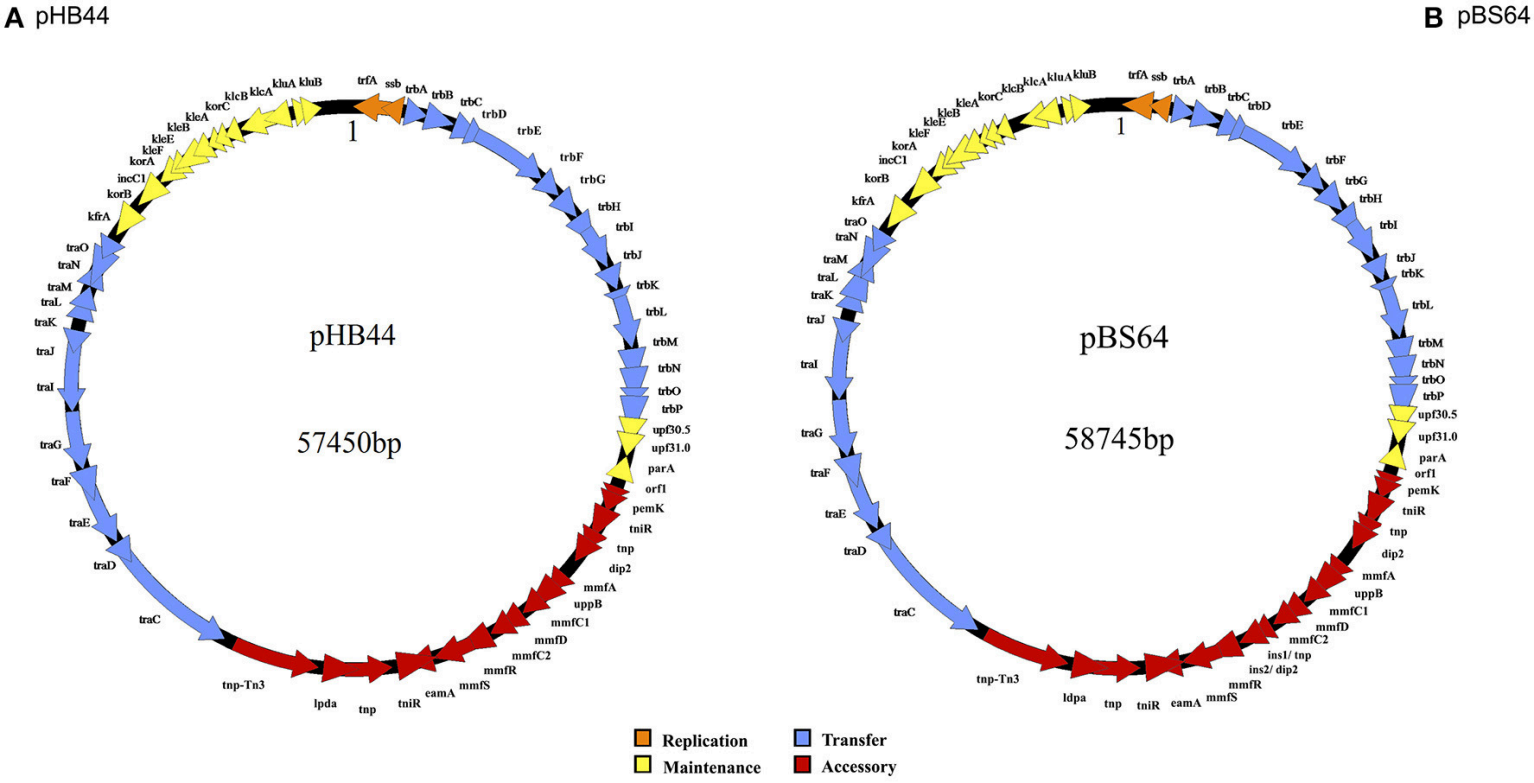
**Circular maps of plasmids pHB44 (A) and pBS64 (B)**. Explanation: 1 represents start of base counting.

The complete nucleotide sequences of plasmids pHB44 and pBS64 were determined by Roche-454 sequencing. Average coverage values were >100, with read lengths of >400 bp. Subsequent computer-assisted as well as manual assemblies (including gap closure using PCR approaches) revealed plasmids pHB44 and pBS64 to be circular, with predicted sizes of 57,450 bp and 58,745 bp, respectively (Figure 1). These sizes were confirmed by using restriction analysis with Sph1 followed by agarose gel electrophoresis. This enzyme was predicted to have 7 and 8 restriction sites in pHB44 and pBS64, respectively, and so produced 8 and 9 bands, respectively, in these plasmids (data not shown). The overall G + C contents of the pHB44 and pBS64 sequences were 64.6 and 64.5%, respectively, which is within the range (64–66%) found across the IncP-1β plasmids. Both plasmids were found to possess replication (evidenced by genes like *trf* A and *ssb*), conjugative DNA transfer (the canonical *tra* gene set), mating-pair formation (*trb* gene set), and stable inheritance and central control regions (*ctl* genes). We found no insertions in the RHS I and RHS III regions in either of the two plasmids, whereas—in both—a suite of accessory genes was found in RHS II (between the *tra* and *trb* regions).

### Annotation of the genomes of plasmids pHB44 and pBS64

Annotation (software-directed and checked manually—gene-by-gene—afterwards) of the pHB44 and pBS64 sequences revealed the existence of 63 and 65 CDSs, with average lengths of 0.815 and 0.807 Kb, respectively. Thus, plasmids pHB44 and pBS64 had 89.44 and 89.32% gene loads, respectively, with—consequently—10.56 and 10.68% intergenic space. Similarity searches showed that most of the CDSs are predicted to encode proteins with high sequence similarities to proteins of a range of other, IncP-1β or related, plasmids. For plasmid pHB44, functions could be attributed to 62 out of the 63 CDSs, with the remaining CDS being described as encoding a hypothetical protein. For plasmid pBS64, 64 of the 65 CDSs were predicted to encode biological functions similar to those of known plasmids, whereas the function of the remaining CDS was unknown. Supplementary Tables S1, S2 list all CDSs, their proposed gene names, localization on the plasmid genome, G+C content, as well as the length and size of the predicted gene products and the similarities to proteins (“best hits”) of GenBank.

### Description and phylogenetic analysis of plasmid backbone structures

The backbones of plasmids pHB44 and pBS64 were highly syntenous and homologous between each other. On the basis of sequence similarity, they were most similar to the backbone of plasmid pMBUI8 (recently obtained from freshwater, Brown et al., 2013), followed by pUO1 and pTP6. Both the pHB44 and pBS64 backbones encompassed about 38 kb of sequence, with 46 predicted CDSs. They consisted of replication (i.e. *trf* A and *ssb*), transfer (*tra* and *trb* genes), and stable inheritance/control (*klc, kle, kor* and *kfr* genes) modules. Specifically, 2 canonical CDSs were predicted to be involved in replication initiation (*trf* A and *ssb*), 16 in mating pair formation (*trb* A through P), 12 in conjugative plasmid transfer (*tra* C through G, I through O) and another 16 in plasmid stabilization, maintenance and control (gene assignments: *parA, klc, kle, kor, kfr, klu, inc, upf30.5, upf31.0*; Figures 1A,B).

To ascertain the phylogenetic placement of plasmids pHB44 and pBS64, trees were constructed using the backbone genes *trb*A, *trb*B, *kor*A, *klc*A, *tra*C, *tra*D and *tra*E, in comparison to a suite of selected IncP-1 plasmids. The data can be found in Supplementary Figures S1a, S1b. Without exception, all trees showed that plasmids pHB44 and pBS64 belong to the IncP-1β plasmid group, and—more specifically—the β-1 subclade. Indeed, very similar tree topologies were observed for all the selected genes, with both new plasmids clustering together with a suite of other typical IncP-1β-1 subgroup plasmids, which invariably included pMBUI8 (Supplementary Figures S1a, S1b).

**Figure 2:**
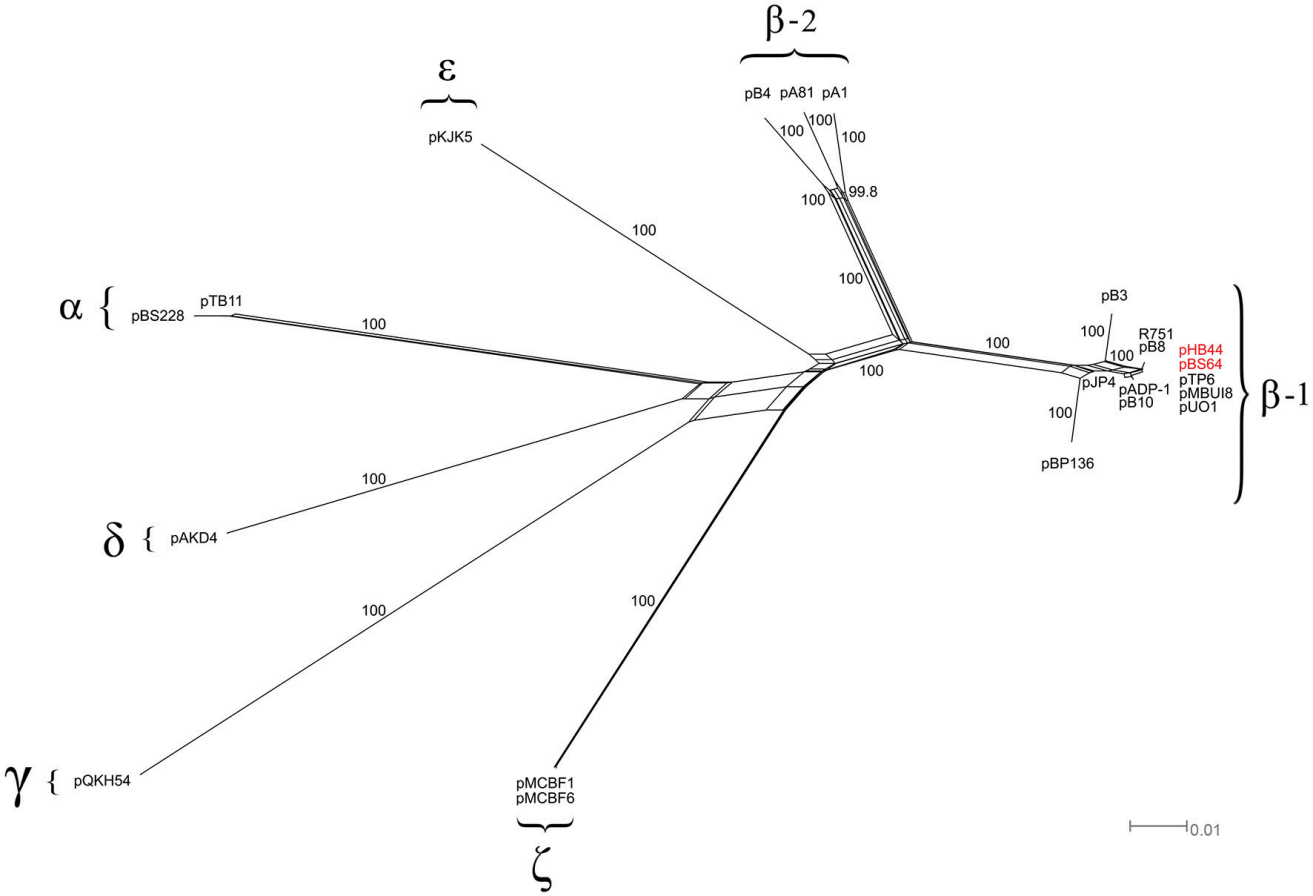
**Phylogenetic analysis of the IncP-1 plasmid backbone (splits-tree approach)**. Phylogenetic network based on the six-gene concatenated regions of 20 IncP-1 plasmids. The network displays seven main clades, including the two newly sequenced plasmids (pHB44 and pBS64, in red).

Then, “splits-tree” networks were constructed on the basis of six backbone genes that were selected in accordance with recent literature, i.e. *trf* A, *trb*A, *kor*A, *klc*A, *tra*C and *tra*G. As shown in Figure 2, a star-shaped phylogeny emerged from this analysis. Both plasmids pHB44 and pBS64 separated out as one distinct branch, as expected, among the β-1 clade of the IncP-1 plasmids, with as their closest relatives plasmids pMBUI8, pUO1 and pTP6.

### Recombinational hot spot II contains an “*mmf*” gene cassette with differential function in plasmids pHB44 and pBS64

**Figure 3:**
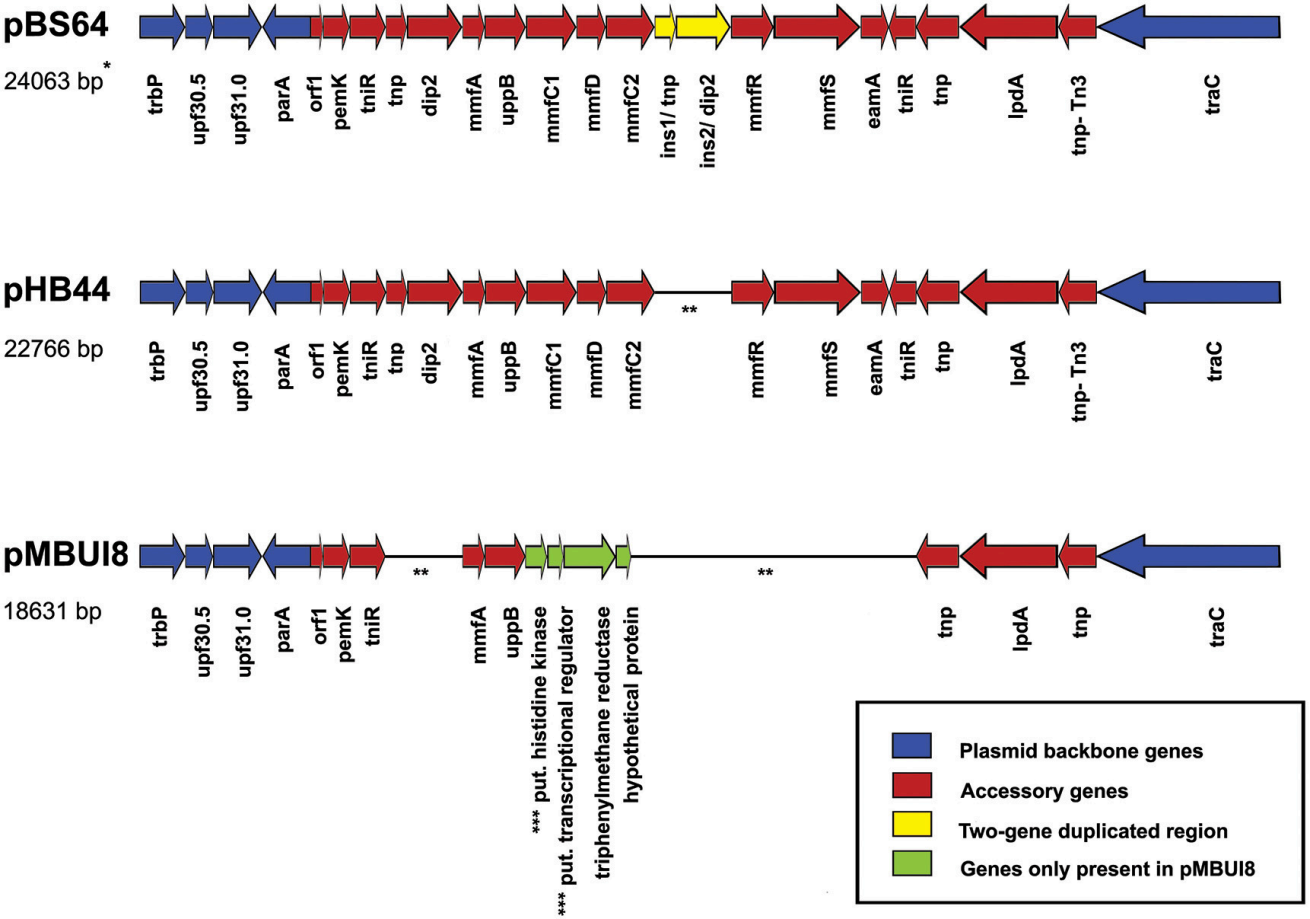
**Comparison of recombinational hot spot II regions between the novel plasmids pBS64 and pHB44 (this paper) and pMBUI8**. Blue, plasmid backbone genes; yellow, two-gene duplicated region; red, accessory genes; green, four pMBUI8 specific genes absent from pBS64 and pHB44. See Table 1 and Supplementary Tables S1, S2 for explanations of the coding regions. ^*^Indicates size including part of the backbone as in figure. ^**^Line is a “connector” (genes are contiguous). ^***^Put.: putative.

Both plasmids pHB44 and pBS64 carried similar-sized accessory gene regions (respectively 15,043 and 16,406 bp for pHB44 and pBS64) in RHS II (between genes *parA* and *tra*C). These inserts matched those of plasmid pMBUI8, albeit only very partially (Figure 3). Across plasmids pHB44 and pBS64, the regions were—to a large extent—similar, revealing the presence of the canonical “*mmf*” (metal response) region that was previously described for the PromA group plasmid pMOL98 (Van der Auwera et al., 2009). The *mmf* regions in the two novel plasmids contained syntenous clusters consisting of the following seven CDSs (at 99–100% of homology): *mmf* A, *mmf* B, *mmf* C1, *mmf* D, *mmf* C2, *mmf* R and *mmf* S, for a total size of 5104 bp in pHB44 and 5105 bp in pBS64. Interestingly, two potential start codons were found in the *mmfB* gene, 45 bp apart. Key aspects explaining the size differences between RHS II of pBS64 and that of pHB44 were the presence in pBS64 of a “two-gene duplicated region”, i.e. *ins1/tnp* and *ins2/dip2*, between the *mmf* C2 and *mmf* R genes. This yielded a 6403 bp total size. Much like in pMOL98, the *mmf* gene cluster was apparently part of a larger transposon, Tn6028, that contained several other functional genes as shown in Figure 3; this is further examined in the Discussion.

### Plasmid-conferred phenotypes—Fe and V uptake and resistance to bacitracin

**Figure 4:**
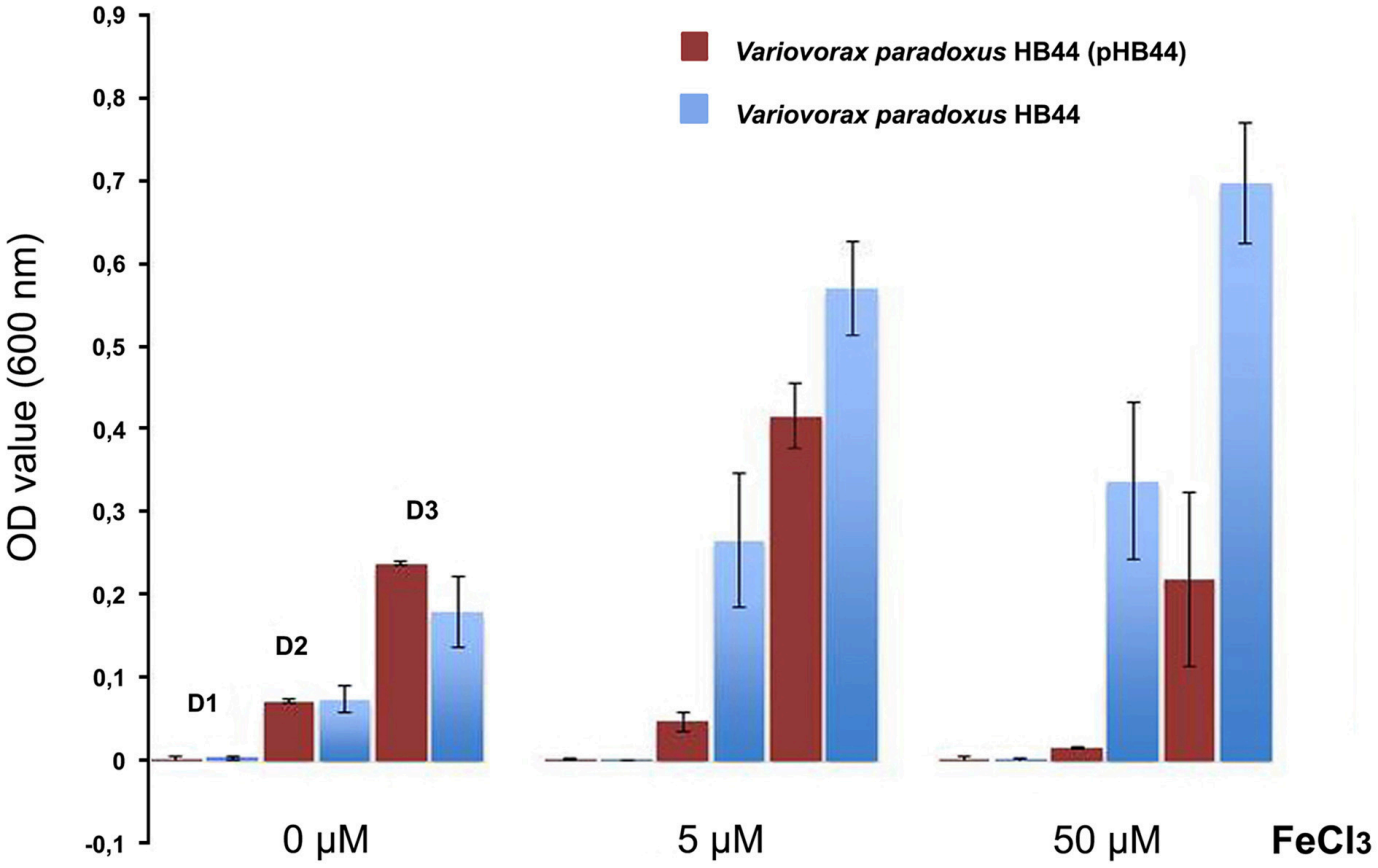
**Phenotype conferred by plasmid pHB44 upon its host**. Growth in M9 mineral medium at three FeCl_3_ levels. Explanation: D0, D2, D3 (valid for all three metal levels) represent OD values at day 1, day 2 and day 3, respectively.

**Figure 5:**
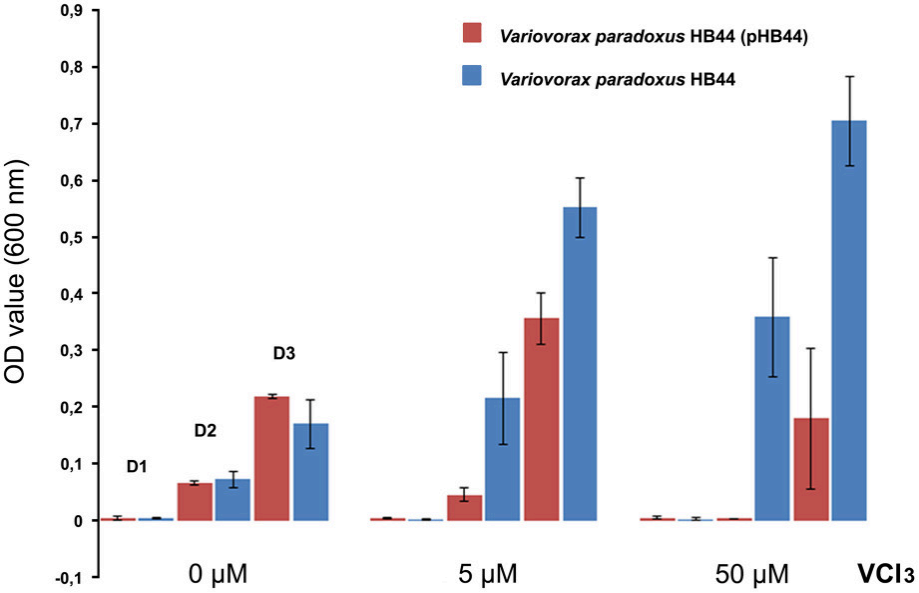
**Phenotype conferred by plasmid pHB44 upon its host**. Growth in M9 mineral medium at three VCl_3_ levels. Explanation: D0, D2, D3 (valid for all three metal levels) represent OD values at day 1, day 2 and day 3, respectively.

To test the potential function of genes of RHS II, including the *mmf* gene cassette, we analyzed the phenotype conferred upon the host. To this end, we selected plasmid pHB44 for pragmatic reasons. First, the potential involvement of plasmid pHB44 in iron uptake (suggested by differences in bacterial survival in the mycosphere under different iron conditions; Zhang et al., 2015) was assessed by studying the growth of plasmid-containing vs. plasmid-cured strains in M9 minimal media with different levels of added FeCl_3_. The data (Figure 4) showed an elevated growth rate of *V. paradoxus* HB44 containing plasmid pHB44 vs. the plasmid-less strain under zero-added-iron conditions. In contrast, it showed significantly lowered growth under 5 and 50 μM iron conditions (*P* < 0.05). Thus, the modulating effect of plasmid pHB44 on the growth /fitness of its host under diverse iron conditions, as previously reported for *V. paradoxus* in the mycosphere, was corroborated by these growth experiments. We then investigated the putative involvement of plasmid pHB44 encoded functions in the uptake of another trivalent metal ion, vanadium. We thus assessed the growth of *V. paradoxus* HB44 (pHB44)—in parallel to plasmid-less HB44—in minimal medium with different levels of VCl_3_. At 0 μM of added VCl_3_, strain HB44 (pHB44) was fitter than its counterpart HB44 in terms of maximal growth (level around 10^7^ CFU/ml; *P* < 0.05), as measured at day 3. In contrast, at 5 μM of VCl_3_, the plasmid-less strain HB44 was consistently “fitter” (as indicated by the population densities reached) than HB44 (pHB44), establishing population densities of 5 × 10^7^ CFU/ml (*P* < 0.05). At 50 μM of VCl_3_, these differences between HB44 (pHB44) and HB44 became even greater (Figure 5).

Finally, growth under progressively increasing bacitracin pressure was assessed in agar media, on the basis of single-colony growth of the plasmid-containing vs. plasmid-cured strains. The data revealed plasmid pHB44 to confer resistance to high concentrations of bacitracin to its host *V. paradoxus* HB44. Whereas the plasmid-cured strain HB44 revealed a MIC of 40 μg/ml bacitracin, strain HB44 (pHB44) had a MIC of 300 μg/ml. In contrast, plasmid pBS64 could not be shown to confer bacitracin resistance on its host, as the plasmid-cured strain BS64 grew up to bacitracin levels of 500 μg/ml, which was similar to strain BS64 (pBS64). Remarkably, control plasmid pMBUI8 which contained two *mmf* genes in RHS II, i.e. *mmfA* and *mmfB* (Figure 3), also conferred resistance to (200 μg/ml) bacitracin to its host, *E. coli* K12. In this case, the plasmid-less control *E. coli* strain had a MIC of 40 μg/ml.

### Functional analysis of recombinational hot spot II

In the RHS II regions of plasmids pHB44 and pBS64, 5104- and 5105- bp *mmf* type gene cassettes were observed. The canonical 5104 bp *mmf* gene cluster, present on plasmid pMOL98, reportedly encodes a “multiple metal response” phenotype in *Cupriavidus metallidurans* (Van der Auwera et al., 2009). As in van der Auwera et al., the cluster was suggested to have been sequestered—presumably by transposition—from the genome of *C. metallidurans* by pMOL98 during the experiments performed by the authors that yielded plasmid pMOL98. Table 1 provides an outline of the genes and their predicted functions present within the confines of RHS II in plasmids pHB44 and pBS64. A two-component sensing/response system, next to genes for several proteins located at/in the cell membrane and for a phosphatidyl phosphatase (*mmf* B) emerged. The *mmfB* gene product may actually be a bcrC-like undecaprenyl pyrophosphate (UPP) phosphatase (Bernard et al., 2005). Next to the discovery of the gene cassette in the two plasmids pHB44 and pBS64, part of it, i.e. genes *mmfA* and *mmfB*, was detected in the closest relative plasmid pMBUI8. Interestingly, this plasmid also conferred a bacitracin resistance to its host.

**Table 1:** **Genes identified in the inserts in recombinational hot spots II (between ***parA*** and ***traC***) of plasmids pHB44 and pBS64, and their putative function^#^**.

Gene (no./name)^*^	**Proposed name**	Size (bp)^**^	Size protein (a.a.)^***^	Transcr. direct^****^	**Amino acid identity to other (plasmid) gene products**	**Predicted function, remarks, reference**
1. *orf1*	*orf1*	216	71	F	100% *Uncultured bacterium plasmid pMBUI8*	Hypothetical; DUF3018 family.
2. *pemK*	*pemK*	321	106	F	89% *Xanthomonas campestris pv. vesicatoria plasmid pXCV38*	Toxin, kills PemI-free cells. In *E. coli*, inhibits growth, resulting in cell death. Together with pemI, constitutes stable maintenance system of plasmid R100.
3. *tniR*	*tniR*	564	187	F	92*% Uncultured bacterium plasmid pKS208*	Resolvase.
4. *tnp*	*tnp*	327	108	F	77% *Ralstonia pickettii plasmid pRp12D02*	Mobile element protein, transposase IS3/ IS911 family.
5. *orf2*	dip2^*****^	852	283	F	79% *Ralstonia pickettii plasmid pRp12D02*	Mobile element protein, HTH domain, ribonuclease H-like domain, integrase. Protein has features of protein fhuA, predicted to bind ferrichrome-metal complexes from milieu.
6. *mmfA*	*mmfA*	336	111	F	99% *Synthetic plasmid pMOL98*, mmfA	Protease inhibitor, PepSY domain. Integral “pepSy” membrane protein, Possibly protection of ferrichrome.
7. *mmfB*	*uppB*	597	198	F	100% *Synthetic plasmid pMOL98*, mmfB	Undecaprenyl pyrophosphate phosphatase. Resembles Cd protein. Confers bacitracin resistance due to UPP activity.
8. *mmfC1*	*mmfC1*	783	260	F	99% *Synthetic plasmid pMOL98*, mmfC1	Integral membrane protein, putative MFS type permease, protein of unknown function DUF347. Also found on pAKD16.
9. *mmfD*	*mmfD*	450	149	F	98% *Synthetic plasmid pMOL98*, mmfD	Secreted protein (Tn6048).
10. *mmfC2*	*mmfC2*	759	252	F	92% *Synthetic plasmid pMOL98*, mmfC2	Integral membrane protein, putative MFS (Tn6048) type permease (DUF 347). Also found on pAKD16.
*-ins1 (pBS64)*	*ins1/tnp*	327	108	F	77% *Ralstonia pickettii 12D plasmid pRp12D02*	Mobile element protein, transposase IS3/IS911 family.
*-ins2 (pBS64)*	*ins2/dip2*	852	283	F	79% *Ralstonia pickettii 12D plasmid pRp12D02*	Mobile element protein, HTH domain, ribonuclease H-like domain, integrase. Protein has features of protein FhuA, predicted to bind ferrichrome-metal complexes from milieu.
11. *mmfR*	*mmfR*	666	221	F	99% *Synthetic plasmid pMOL98*, mmfR	Two-component transcriptional regulator, CheY-like superfamily.
12. *mmfS*	*mmfS*	1353	450	F	100% *Synthetic plasmid pMOL98*, mmfS	Two-component sensor, periplasmic signal transduction histidine kinase (Tn6048).
13. *orf3*	*eamA*	432	143	F	100% *Cupriavidus metallidurans CH34 megaplasmid*, conserved hypothetical protein	Membrane protein with (drug) transport function. Has eamA domain, many members of this family are classed as drug/metabolite transporters.
14. *tniR*	*tniR*	669	222	R	99% *Cupriavidus metallidurans CH34 megaplasmid*, Tn6048 tniR	Resolvase.
15. *tnp*	*tnp*	1548	515	R	97% *Comamonas* sp. *plasmid pKV36 accessory region*	Transposase IS801/ IS1294, zinc-binding domain.
16. *lpdA*	*lpdA*	591	196	R	93% *Uncultured bacterium plasmid pMBUI8*, dihydrolipoamide dehydrogenase	FAD/NAD-linked reductase, pyridine nucleotide-disulphide oxidoreductase, dihydrolipoamide dehydrogenase.
17*. tnp*^∧^	*tnp-Tn3*	2850/2916	949/971	R	99% *Ralstonia eutropha JMP134 plasmid pJP4*, tnpA	Mobile element protein, Tn3 family transposase, domain of unknown function DUF4158.

# *Gene count starts after gene parA and ends before traC*.

* *Name taken from literature and proposed here on the basis of presumed function*.

** *Stop codon included*.

*** *a.a: number of amino acids*.

**** *Transcr. direct: direction of transcription (F: forward, R: reverse)*.

***** *dip2: DNA-interactive protein 2*.

∧ *Size for pHB44/ size for pBS64*.

## Discussion

In this study, we provide evidence for the contention that the *L. proxima* mycosphere (in Gieterveen soil) harbors *Variovorax* spp. that possess mobile traits with roles in the responses to metals and/or bacitracin. First, our data on plasmid prevalence in *Variovorax* isolates across three habitats appeared to corroborate the contention that plasmids are selected for in the Gieterveen soil *L. proxima* mycosphere, as their prevalence was high (17%) across *Variovorax* strains from the aforementioned mycosphere vs. zero across those from the two comparator (rhizosphere) habitats. Thus, the presence of intermediate-sized plasmids like pHB44 and pBS64 is assumed to have significance for the ecological plasticity of *Variovorax* spp. in the former habitat. Analysis of the complete nucleotide sequences of selected plasmids might thus shed light on potential plasmid-encoded fitness-modulating traits. On the basis of accepted genome-based criteria, the two plasmids were classified as typical IncP-1β-1 plasmids, with as their closest relative plasmid pMBUI8. The conservation of both the homology and synteny of the plasmid backbone modules across these three plasmids, as well as a larger suite of plasmids, is noteworthy, as it points to functional/organizational constraints that tend to keep the currently known suite of IncP-1β-1 plasmids almost clonal in their backbones. Sen et al. (2011) defined the two subclades of IncP-1β plasmids, that are different in the plasmid backbone gene sequence as well as in the gene content within the *ctl* region (*kfr*C to *klc*A). They suggested that the IncP-1β-1 and -2 clades have undergone extensive recombinations (Sen et al., 2012). Here, we conclude that the two novel plasmids pHB44 and pBS64 actually show little evidence for recombinations across their backbones. A remarkable finding was that the toxin gene pemK was present yet no homolog of the antitoxin pemI was found. We cannot explain this apparent lack of a full stability system, but suggest that either pemK was dysfunctional or a substitute for pemI, e.g. orf1, was present. However, we examined the orf 1 sequence (amino acid level) for homology to a known pemI protein, but found a very low homology value.

Next to their conserved backbones, we also observed a lack of insertions in both plasmids in RHS I and RHS III. In contrast, 15,043 and 16,406-kb insertions were found in the RHS II regions of pHB44 and pBS64, with part of these similar to the inserted region present in plasmid pMBUI8. In this respect, the finding of the canonical *mmf* gene cassette as carried by transposon Tn6048 in plasmids pHB44 and pBS64 indicated the occurrence of (1) transpositional activity in this region in the mycosphere/soil setting, and (2) potential selective pressure favoring such insertions vs. the locus devoid of Tn6048 and *mmf*. In this respect, we cannot exclude the possibility of plasmid-to-chromosome/chromosome-to-plasmid “traffic” of the transposon, or even the whole plasmid. The canonical *mmf* gene cluster reportedly encodes a “multiple metal response” phenotype in *C. metallidurans* (Van der Auwera et al., 2009). However, in spite of testing a suite of 16 metals (excluding Fe and V), the authors did not provide great detail about the functioning of this gene cassette. We thus investigated the possible phenotype encoded by *mmf* on plasmids pHB44 and pBS64. Regarding plasmid pHB44, we previously obtained experimental evidence for the contention that it can assist its host with Fe acquisition in the mycosphere, under Fe-limiting conditions (Zhang et al., 2015). We here confirm this iron-capturing effect also for bacterial cells grown in minimal media, and extend the effect to the capturing of the essential metal vanadium. Although under debate, vanadium may have a role as a component of peroxidases, and it is known to accumulate in some soil fungi (Simons et al., 1995). In our experiments with Fe and V, we used common glassware, reagents and demineralized water. Although it is possible that additional Fe and V ions entered the media used, we argue that this will be the case across all treatments. Hence, the relative effects of plasmid pHB44 carriage, as detected by us, should be interpreted, with the cautionary note in mind, that the final iron levels may have digressed from the established ones. Moreover, the fitness-decreasing effect of the plasmid might also be related to toxicity by the ions taken up by the capturing system, as previously discussed in Zhang et al. (2015).

The transition element vanadium (atomic number 23) is chemically similar to iron (atomic number 26) in that it also has 2+ and 3+ valency states. We here hypothesize, on the basis of a molecular analysis of the *mmf* gene cassette sequence (Table 1), that this system, regulated by a two-component sensing/response system (encoded by *mmfR* and *mmfS*, respectively; Figure 3) is involved in the facilitation of capturing of ferrichrome-bound metals like iron and vanadium from the environment by the *V. paradoxus* host. Ferrichromes are cyclic metal (iron)—binding hexapeptides (derived from ornithine) that are often produced by soil fungi, in particular in acid soils. Given this fact, we cogitated that the possession of a “metal uptake facilitator” system on plasmid pHB44 endows *V. paradoxus* HB44 with a fitness-enhancing trait in conditions of competition for scarce iron. Although not proven, it is an intriguing thought that this system may allow the bacterium to capture, next to “self” iron-loaded ferrichromes, fungal-released ones as well. Our finding of enhanced resistance to bacitracin conferred by plasmids pHB44 as well as pMBUI8 on their hosts placed a focus on the *mmf* B gene. The *mmfB* gene product may actually be a bcrC-like undecaprenyl pyrophosphate phosphatase (UPP), as found in BLAST-X analyses. Bernard et al. (2005) described that bacitracin bound to metal(s) can bind to UPP, preventing its dephosphorylation into undecaprenyl phosphate (UP) and thus hampering “normal” cell wall synthesis (UP is the lipid carrier essential for peptidoglycan synthesis). Thus, sequestering UPP away to UP is supposed to “compete” with bacitracin binding, safeguarding the normal cell wall synthesis of the organism confronted with bacitracin pressure.

What benefit would a metal capturing system together with a bacitracin resistance on a plasmid in Gieterveen soil/mycosphere have? In the light of the fact that bacitracin is produced by soil bacilli as a small (8–10 amino acids) oligopeptide that has metal-binding properties, we surmised that soil/mycosphere-released bacitracin might be inadvertently captured by the efficient metal uptake system, thus resulting in the consequential hampering of bacterial cell wall synthesis. Hence, the UPP protein produced by *mmfB* might actually work like a “rescuing” system, allowing the system to work without detriment to host ecological fitness.

The finding of the total *mmf* gene cassette on the broad-host-range self-transferable IncP-1β1 plasmids pHB44 and pBS64 was revealing, as it points to a scenario of sequestration of genes from another (soil) organism, followed by selection of the plasmid/host combination under prevailing conditions, under which iron and/or vanadium limitation and potential bacitracin pressure. This might have been followed by subsequent transfer of the plasmid into a host with superior ecological competence in the mycosphere.

This is the first time that the *mmf* gene cassette—in transposon Tn6048—was found on an IncP-1β plasmid, indicating the key role of transposition next to plasmid transfer as processes that allow gaining superior mycosphere competence (Zhang et al., 2014a). With respect to plasmid pBS64, previous soil microcosm experiments did not reveal a strong additional iron uptake function. Moreover, we could not discern a clear bacitracin resistance, as the host was already resistant to high bacitracin levels. It is intriguing to note that a two-gene duplicated region was found between the *mmf* C2 and *mmf* R genes on pBS64. Thus, pBS64, which included genes no. 4 (*tnp*) and 5 (*dip2*) (Table 1). may constitute the serendipitously-found “control” of the traits shown by functional plasmid pHB44. However, the exact machinery underlying the lack of detectable effect is still unknown.

## Author contributions

JDvE conceived of the experiments and contributed to the article writing. MZ performed the experiments and contributed to the article writing. JKB was involved in experiments, performed data analysis and contributed to the article writing.

### Conflict of interest statement

The authors declare that the research was conducted in the absence of any commercial or financial relationships that could be construed as a potential conflict of interest.

## Supplementary material

Supplementary Figures S1a, S1b **Phylogenetic trees using the backbone genes**. trbA (A), trbB (B), traC (C), traD (D), traE (E), klcA (F), and korC (G) (plasmids pHB44 and pBS64 in red).

Supplementary Table S1 **Putative coding regions of plasmid pHB44 and their predicted functions**.

Supplementary Table S2 **Putative coding regions of plasmid pBS64 and their predicted functions**.
