# Supplementary figures and images for: The Complete Sequences and Ecological Roles of Two IncP-1β Plasmids, pHB44 and pBS64, Isolated from the Mycosphere of Laccaria proxima
Source: Front Microbiol. 2016 Jun 21;7:909. doi: 10.3389/fmicb.2016.00909 (PMC4914505; doi:10.3389/fmicb.2016.00909)

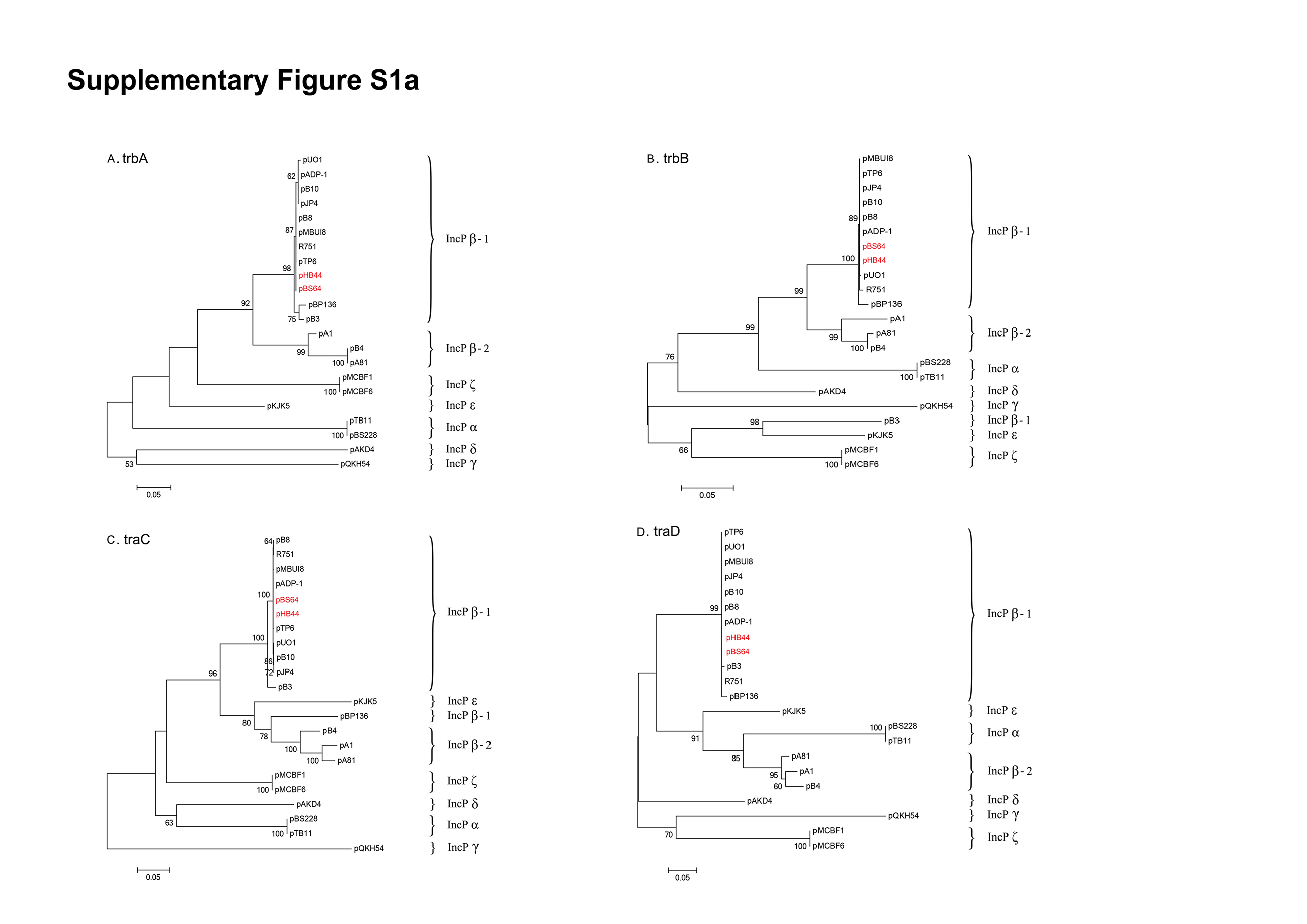

Supplement: Supplementary Figures S1a, S1b — Phylogenetic trees using the backbone genes. trbA (A), trbB (B), traC (C), traD (D), traE (E), klcA (F), and korC (G) (plasmids pHB44 and pBS64 in red). [file Image1.tif]

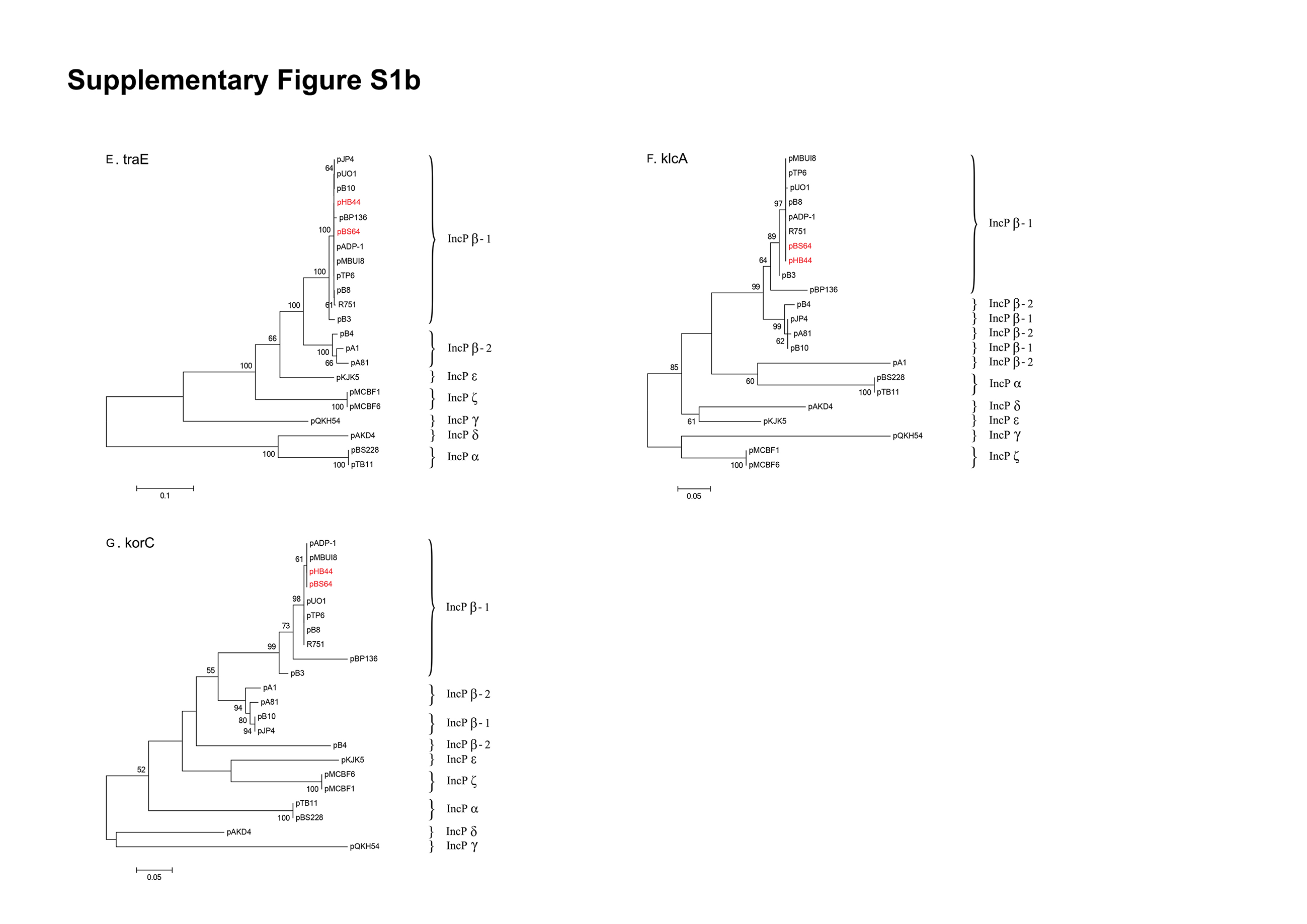

Supplement: Supplementary file 2 [file Image2.tif]
