# Supplementary material for: The Complete Sequences and Ecological Roles of Two IncP-1β Plasmids, pHB44 and pBS64, Isolated from the Mycosphere of Laccaria proxima
Source: Front Microbiol. 2016 Jun 21;7:909. doi: 10.3389/fmicb.2016.00909 (PMC4914505; doi:10.3389/fmicb.2016.00909)
Supplement: Supplementary Table S1 — Putative coding regions of plasmid pHB44 and their predicted functions. [file Table1.pdf]

Supplementary Table S1. Putative coding regions of plasmid pHB44 and their predicted functions.

CDS	Start position	Stop position	Length (bp)	G+C content (%)	Predicted Function	Protein length (aa)	Protein size (kDa)	Identity (%) to: pMBUI8	R751
trfA	1221	1	1221	65	replication initiation protein	406	45.8	100	99
ssb	1609	1268	342	60	primosome PriB, single-strand DNA-binding	113	12.7	100	100
trbA	1723	2085	363	59	conjugative transfer protein	120	13.3	100	100
trbB	2395	3357	963	62	conjugative transfer protein, ATPase	320	35.1	100	99
trbC	3374	3838	465	67	conjugative transfer protein, prepilin	154	16.4	100	99
trbD	3842	4153	312	63	conjugative transfer protein	103	11.8	100	99
trbE	4150	6708	2559	64	conjugative transfer protein	852	94.0	100	99
trbF	6705	7487	783	66	conjugative transfer protein	260	28.7	100	99
trbG	7505	8404	900	64	conjugative transfer protein	299	32.7	100	99
trbH	8407	8904	498	71	conjugative transfer protein	165	17.6	99	99
trbI	8901	10322	1422	67	conjugative transfer protein	473	49.4	100	99
trbJ	10343	11107	765	61	conjugative transfer protein	254	27.5	100	99
trbK	11117	11344	228	62	conjugative transfer entry exclusion protein	75	8.0	100	99
trbL	11346	13073	1728	68	conjugative transfer protein	575	56.2	100	99
trbM	13091	13678	588	67	conjugative transfer protein	195	21.6	100	100
trbN	13692	14327	636	69	conjugative transfer protein	211	22.8	99	99
trbO	14356	14622	267	63	conjugative transfer protein	88	9.9	99	99
trbP	14622	15320	699	68	conjugative transfer protein	232	25.7	100	99
upf30.5	15336	15767	432	66	outer membrane protein, major outer membrane lipoprotein,	143	15.1	100	99

					S-adenosyl-L-methionine-dependent				
upf31.0	15838	16596	759	65	major outer membrane protein, methyltransferase	252	28.3	99	100
parA	17353	16598	756	70	resolvase	251	27.4	100	-
orf1	17642	17857	216	62	hypothetical protein, protein of unknown function DUF3018	71	8.2	100	-
pemK	17857	18177	321	56	mRNA interferase PemK-like protein, plasmid maintenance toxin/ cell growth inhibitor	106	11.6	100	-
tniR	18413	18976	564	68	resolvase	187	20	100	-
tnp	19239	19565	327	59	mobile element protein, transposase IS3/ IS911 family protein	108	11.8	-	-
dip2	19580	20431	852	60	mobile element protein, HTH-like domain, ribonuclease H-like domain, integrase, catalytic region. Protein has features of membrane protein fhuA, predicted to bind ferrichrome-metal complexes from milieu.	283	33.0	-	-
mmfA	20976	21311	336	62	integral membrane protein, propeptide, pepSy, peptidase M4	111	11.9	100	-
uppB	21368	21964	597	59	Undecaprenyl pyrophosphate phosphatase. Resembles Cd protein. Confers bacitracin resistance due to UPP activity.	198	21.8	100	-
mmfC1	22035	22817	783	62	integral membrane protein, hypothetical protein, putative permease, protein of unknown function DUF347	260	28.1	-	-
mmfD	22847	23296	450	59	secreted protein (Tn6048)	149	15.4	-	-
mmfC2	23306	24064	759	63	integral membrane protein, putative permease of the Major Facilitator Superfamily (Tn6048), protein of unknown function DUF347	252	26.5	-	-
mmfR	24087	24752	666	62	two component transcriptional regulator, signal transduction response regulator, CheY-like superfamily	221	23.8	-	-
mmfS	24728	26080	1353	60	signal transduction histidine kinase (Tn6048)	450	49.2	-	-
eamA	26109	26540	432	56	putative membrane protein TN6048, eamA domain	143	15.3	-	-
tniR	27275	26607	669	62	resolvase	222	23.9	-	76

tnp	28988	27441	1548	64	transposase IS801/ IS1294, transposase zinc binding domain	515	56.6	98	-
ldpA	29689	29099	591	66	FAD/NAD-linked reductase, pyridine nucleotide-disulphide	196	20.2	93	-
					oxidoreductase, dihydrolipoamide dehydrogenase				
tnp-Tn3	32685	29836	2850	60	mobile element protein, Tn3 family transposase,	949	106.1	99	-
					domain of unknown function DUF4158				
traC	37388	33042	4347	69	DNA processing protein, DNA primase	1448	159.0	99	99
traD	37781	37392	390	72	DNA transfer protein	129	13.6	99	99
traE	39866	37803	2064	67	DNA processing protein, DNA topoisomerase III	687	74.9	100	99
traF	40414	39878	537	66	DNA transfer maturation peptidase protein	178	18.9	100	99
traG	42324	40411	1914	66	DNA transfer coupling protein	637	69.9	99	99
traI	44561	42321	2241	67	DNA relaxase protein	746	82.1	100	99
traJ	44970	44596	375	67	oriT binding protein	124	14.2	100	100
traK	45344	45742	399	63	oriT binding protein	132	14.6	100	99
traL	45742	46467	726	63	DNA transfer protein	241	26.4	100	99
traM	46467	46907	441	68	DNA transfer protein	146	15.6	100	100
traN	47763	47110	654	69	DNA transfer protein	217	24.1	100	99
traO	48139	47792	348	58	DNA transfer protein	115	12.5	100	99
kfrA	49341	48310	1032	75	regulatory protein	343	36.8	100	99
korB	50570	49521	1050	67	transcriptional repressor protein, plasmid partitioning protein,	349	38.2	100	99
					parB/ Sulfiredoxin				
incC1	51331	50567	765	64	partitioning protein, P-loop containing nucleoside triphosphate hydrolase,	254	27.7	100	100
					cobQ/ cobB/ minD/ parA nucleotide binding domain				
korA	51630	51328	312	62	transcriptional repressor protein	100	11.0	100	100
kleF	52274	51744	531	69	protein of unknown function DUF2761	176	18.7	100	99

kleE	52605	52276	330	65	stable inheritance protein	109	12.0	100	100
kleB	52965	52750	216	63	protein of unknown function DUF2688, possible transcriptional regulation	71	7.7	100	100
kleA	53260	53024	237	64	stable inheritance protein	78	8.6	100	100
korC	53677	53420	258	70	transcriptional repressor protein	85	9.3	100	99
klcB	54899	53694	1206	70	stable inheritance protein	401	42.7	99	99
klcA	55379	54951	429	69	antirestriction protein	142	15.7	100	100
kluA	55536	55817	270	67	addiction module antitoxin protein, uncharacterised protein family UPF0156/ metJ/ arc repressor	93	10.2	100	100
kluB	55814	56128	315	65	addiction module toxin protein, MSL3420 protein, toxin-antitoxin system, relE/ parE toxin family	104	11.6	100	99
