# Supplementary material for: The Complete Sequences and Ecological Roles of Two IncP-1β Plasmids, pHB44 and pBS64, Isolated from the Mycosphere of Laccaria proxima
Source: Front Microbiol. 2016 Jun 21;7:909. doi: 10.3389/fmicb.2016.00909 (PMC4914505; doi:10.3389/fmicb.2016.00909)
Supplement: Supplementary Table S2 — Putative coding regions of plasmid pBS64 and their predicted functions. [file Table2.pdf]

Supplementary Table S2. Putative coding regions of plasmid pBS64 and their predicted functions.

**CDS**	**Start position**	**Stop position**	**Length (bp)**	**G+C content (%)**	**Predicted Function**	**Protein length (aa)**	**Protein size (kDa)**	**Identity (%) to:**	
								**pMBUI8**	**R751**
trfA	1029	1	1029	65	replication initiation protein	342	39.1	100	99
ssb	1608	1267	342	60	primosome PriB, single-strand DNA-binding	113	12.7	100	100
trbA	1722	2084	363	59	conjugative transfer protein	120	13.3	100	100
trbB	2394	3356	963	62	conjugative transfer protein, ATPase	320	35.1	100	99
trbC	3373	3837	465	67	conjugative transfer protein, prepilin	154	16.4	100	99
trbD	3841	4152	312	63	conjugative transfer protein	103	11.8	100	99
trbE	4149	6707	2559	64	conjugative transfer protein	852	94.0	100	99
trbF	6704	7486	783	66	conjugative transfer protein	260	28.7	100	99
trbG	7504	8403	900	64	conjugative transfer protein	299	32.7	100	99
trbH	8406	8894	489	71	conjugative transfer protein	162	17.0	100	99
trbI	8899	10320	1422	67	conjugative transfer protein	473	49.4	100	99
trbJ	10341	11105	765	61	conjugative transfer protein	254	27.5	100	99
trbK	11115	11342	228	62	conjugative transfer entry exclusion protein	75	8.0	100	99
trbL	11353	13071	1719	68	conjugative transfer protein	572	55.9	100	99
trbM	13089	13676	588	67	conjugative transfer protein	195	21.6	100	100
trbN	13690	14325	636	69	conjugative transfer protein	211	22.8	99	99
trbO	14354	14620	267	63	conjugative transfer protein	88	9.9	99	99
trbP	14620	15318	699	68	conjugative transfer protein	232	25.7	100	99
upf30.5	15334	15765	432	66	outer membrane protein, major outer membrane lipoprotein,	143	15.1	100	99

					S-adenosyl-L-methionine-dependent				
upf31.0	15836	16594	759	65	major outer membrane protein, methyltransferase	252	28.4	99	99
parA	17351	16596	756	70	resolvase	251	27.4	100	-
orf1	17640	17855	216	62	hypothetical protein, protein of unknown function DUF3018	71	8.2	100	-
pemK	17855	18175	321	56	mRNA interferase PemK-like protein, plasmid maintenance toxin/ cell growth inhibitor	106	11.6	100	-
tniR	18411	18974	564	68	resolvase	187	20.0	100	-
tnp	19237	19563	327	59	mobile element protein, transposase IS3/ IS911 family protein	108	11.8	-	-
dip2	19578	20429	852	60	mobile element protein, HTH-like domain, ribonuclease H-like domain, integrase, catalytic region. Protein has features of membrane protein fhuA, predicted to bind ferrichrome-metal complexes from milieu.	283	33.0	-	-
mmfA	20974	21309	336	62	integral membrane protein, propeptide, pepSy, peptidase M4	111	11.9	100	-
uppB	21321	21962	642	59	Undecaprenyl pyrophosphate phosphatase. Resembles Cd protein. Confers bacitracin resistance due to UPP activity.	213	23.4	100	-
mmfC1	22033	22815	783	62	integral membrane protein, hypothetical protein, putative permease, protein of unknown function DUF347	260	28.1	-	-
mmfD	22845	23294	450	59	secreted protein (Tn6048)	149	15.4	-	-
mmfC2	23304	24062	759	63	integral membrane protein, putative permease of the Major Facilitator Superfamily (Tn6048), protein of unknown function DUF347	252	26.5	-	-
ins1/ tnp	24152	24478	327	59	mobile element protein, transposase IS3/ IS911 family protein	108	11.8	-	-
ins2/ dip2	24493	25344	852	60	mobile element protein, HTH-like domain, ribonuclease H-like domain, integrase, catalytic region. Protein has features of membrane protein fhuA, predicted to bind ferrichrome-metal complexes from milieu.	283	33.1	-	-
mmfR	25384	26049	666	62	two component transcriptional regulator, signal transduction response regulator,	221	23.9	-	-

					CheY-like superfamily				
mmfS	26025	27377	1353	60	signal transduction histidine kinase (Tn6048)	450	49.2	-	-
eamA	27406	27837	432	56	putative membrane protein TN6048, eamA domain	143	15.3	-	-
tniR	28572	27904	669	62	resolvase	222	23.9	-	76
tnp	30285	28738	1548	64	transposase IS801/ IS1294, transposase zinc binding domain	515	56.6	98	-
ldpA	30984	30394	591	66	FAD/NAD-linked reductase, pyridine nucleotide-disulphide oxidoreductase, dihydrolipoamide dehydrogenase	196	20.2	93	-
tnp-Tn3	34046	31131	2916	60	mobile element protein, Tn3 family transposase, domain of unknown function DUF4158	971	108.7	99	-
traC	38683	34337	4347	69	DNA processing protein, DNA primase	1448	159.0	99	99
traD	39076	38687	390	72	DNA transfer protein	129	13.6	99	99
traE	41161	39098	2064	67	DNA processing protein, DNA topoisomerase III	687	74.9	100	99
traF	41709	41173	537	66	DNA transfer maturation peptidase protein	178	18.9	100	99
traG	43619	41706	1914	66	DNA transfer coupling protein	637	69.9	99	99
traI	45856	43616	2241	67	DNA relaxase protein	746	82.1	100	99
traJ	46265	45891	375	67	oriT binding protein	124	14.2	100	100
traK	46639	47037	399	63	oriT binding protein	132	14.6	100	99
traL	47037	47762	726	63	DNA transfer protein	241	26.4	100	99
traM	47762	48202	441	68	DNA transfer protein	146	15.6	100	100
traN	49058	48405	654	69	DNA transfer protein	217	24.1	100	99
traO	49434	49087	348	58	DNA transfer protein	115	12.5	100	99
kfrA	50636	49605	1032	75	regulatory protein	343	36.8	100	99
korB	51865	50816	1050	67	transcriptional repressor protein, plasmid partitioning protein, parB/ Sulfiredoxin	349	38.2	100	99

incC1	52626	51862	765	64	partitioning protein, P-loop containing nucleoside triphosphate hydrolase, cobQ/ cobB/ minD/ parA nucleotide binding domain	254	27.7	100	100
korA	52925	52623	303	62	transcriptional repressor protein	100	11.0	100	100
kleF	53569	53039	531	69	protein of unknown function DUF2761	176	18.7	100	99
kleE	53900	53571	330	65	stable inheritance protein	109	12.0	100	100
kleB	54260	54045	216	63	protein of unknown function DUF2688, possible transcriptional regulation	71	7.7	100	100
kleA	54555	54319	237	64	stable inheritance protein	78	8.6	100	100
korC	54972	54715	258	70	transcriptional repressor protein	85	9.3	100	99
klcB	56194	54989	1207	70	stable inheritance protein	283	29.4	99	99
klcA	56674	56246	429	69	antirestriction protein	142	15.7	100	100
kluA	56831	57112	282	67	addiction module antitoxin protein, uncharacterised protein family UPF0156/ metJ/ arc repressor	93	10.2	100	100
kluB	57109	57423	315	65	addiction module toxin protein, MSL3420 protein, toxin-antitoxin system, relE/ parE toxin family	104	11.6	100	99
